# Living Alone Is Not Associated With Cardiovascular Events and Hypoglycemia in Patients With Type 2 Diabetes Mellitus

**DOI:** 10.3389/fpubh.2022.883383

**Published:** 2022-05-20

**Authors:** Zhaowei Zhu, Zhenyu Peng, Zhenhua Xing

**Affiliations:** ^1^Department of Cardiovascular Medicine, The Second Xiangya Hospital, Central South University, Changsha, China; ^2^Department of Emergency Medicine, Second Xiangya Hospital, Central South University, Changsha, China; ^3^Emergency Medicine and Difficult Diseases Institute, Central South University, Changsha, China

**Keywords:** living alone, hypoglycemia requiring any assistance, hypoglycemia requiring medical assistance, major cardiovascular events, type 2 diabetes mellitus

## Abstract

**Objective:**

Living alone is often associated with reduced social support. However, there are limited data on the relationship between living alone and cardiovascular events or hypoglycemia in patients with type 2 diabetes mellitus (T2DM). This study reports a *post-hoc* analysis of the “Action to Control Cardiovascular Risk in Diabetes (ACCORD)” study.

**Research Design and Methods:**

The Cox proportional hazard models were used to compare the hazard ratios (HRs) for the adverse health events selected as primary endpoints in the study participants; these were compared between those living alone and those living with others. The primary outcomes were hypoglycemia requiring any assistance (HAA), hypoglycemia requiring medical assistance (HMA), and major cardiovascular events (MACEs, including cardiac death, non-fatal myocardial infarction (MI), and non-fatal stroke). Our study included 10,249 participants (2,078 living alone) with a follow-up period of 4.91 ± 1.22 years.

**Results:**

After a multivariable adjustment, the risk of HAA, HMA, and MACEs did not differ significantly between participants living alone and those living with others (HAA, HR: 0.88, 95% CI: 0.75–1.04, *P* = 0.13; HMA, HR: 1.11, 95% CI: 0.92–1.34, *P* = 0.26; MACEs, HR: 0.98, 95% CI: 0.80–1.19, *P* = 0.82). Participants living alone had higher levels of glycated hemoglobin in the middle follow-up period than those living with others.

**Conclusions:**

In patients with T2DM, living alone did not increase the risk of cardiovascular events (cardiac death, non-fatal MI, or non-fatal stroke) and hypoglycemia. Patients living alone had higher Hb1AC levels than those living with others. Clinicians should consider an effective blood glucose control regardless of their living arrangement.

## Background

The number of single-person households among elderly individuals is continually increasing among elderly persons in developed countries such as the United States (1, 2); this has become an important social issue in rapidly aging societies. This is shown by the increase in the proportion of single-person households from 7.7% in 1940 to 25.8% in 2000 in the United States (3). Living alone may lead to social isolation and a lack of social support, which may be detrimental to health (4). This phenomenon was verified in patients with cardiovascular disease (CVD) (5, 6). These studies on living alone and adverse cardiac events have primarily been investigated in patients with CVD, and only few of these studies have focused on type 2 diabetes mellitus (T2DM). However, T2DM is becoming a global health problem, closely leading to adverse cardiovascular events (7). Therefore, for patients with T2DM, the association between living alone and CVD requires further research.

Patients with T2DM must perform home glucose monitoring and adjust their own glucose-lowering medications accordingly. The complexity of many medications may lead to treatment-related adverse events, such as hypoglycemia. The support of others may be an important determinant of the risk of hypoglycemia related to medications (8). Furthermore, the role of family members in moderating and normalizing the disruptive effects of diabetes mellitus is well recognized, and family members often play an instrumental role in helping with detection and treatment of hypoglycemia (9), although very little is known about whether living alone increases the risk of CVD and hypoglycemia. This study evaluated whether living alone increases the risk of hypoglycemia and CVD among patients with T2DM.

## Methods

This study was a *post-hoc* analysis of the “Action to Control Cardiovascular Risk in Diabetes” (ACCORD) study, whose design and outcomes have been described previously (10, 11). The ACCORD study had a double 2 × 2 factorial design and included 10,251 patients with T2DM aged 40–79 years, with a median diabetes duration of 10 years, glycated hemoglobin (HbA1C) levels ≥ 7.5%, and high-risk features for CVD. The ACCORD study aimed to evaluate whether intensified control of blood glucose, blood pressure, or lipid levels could improve CVD outcomes. All participants were randomly assigned to receive either intensive blood glucose control with the aim of achieving HbA1C <6.0% or a standard strategy with the aim of keeping the HbA1C levels at 7.0–7.9%. The participants were recruited from 77 clinical centers in the United States and Canada. All the participants received lifestyle and diabetes management education. Furthermore, all the participants were assigned to either a blood pressure trial or lipid trial. After a follow-up duration of averagely 3.5 years, the intensified glucose control strategy was stopped because this strategy was found to increase the risk of all-cause mortality (11). The participants of each of the trials were followed up at least every 4 months until the end of the trial. Thus, the data on the primary outcomes [a composite of non-fatal myocardial infarction (MI), non-fatal stroke, or cardiac death] was collected and analyzed for an additional 17 months by a central committee, whose members were unaware of the study group assignments (11).

The baseline characteristics of the participants were collected, including demographics, medications, and laboratory test results. Participants living with one or more other adults were considered living with others; otherwise, they were designated as those living alone. At each visit, the participants were asked about their experiences of “low blood sugar.” Severe hypoglycemia was defined as participants with a blood glucose <2.8 mmol/d or have symptoms of hypoglycemia that resolved promptly with oral carbohydrates, intravenous glucose, or parenteral glucagon. Hypoglycemia requiring medical assistance (HMA) was defined as episodes requiring hospitalization or care by emergency response personnel. Hypoglycemia requiring any assistance (HAA) was defined as episodes of hypoglycemia requiring other third-party assistance (12). This study analyzed the time from randomization until the first episode of HAA and/or HMA and the aforementioned primary outcomes. The individual components of major cardiovascular events (MACEs), including cardiac death, non-fatal MI, and non-fatal stroke, were considered the second primary endpoints for this study.

The baseline characteristic data for the included participants are presented as numbers, proportions, or means ± SDs. Less than 1% of the participants had missing values; these participants were excluded. The included participants were divided into two groups according to their living status: living alone or living with others, as described previously. Continuous variables were compared using the Student's *t*-test or the Mann–Whitney U tests according to the distribution type. Categorical variables were compared using chi-square tests. The Kaplan–Meier survival curves were constructed for the selected health events in participants who lived alone compared to those in participants living with others. Cox proportional hazard models were used to compare hazard ratios (HRs) for the selected health events between participants living alone and those living with others. Multivariate adjustments were made to investigate whether other known lifestyle, socioeconomic, and cardiovascular risk factors could explain the association between those living alone and the risk of CVD and hypoglycemia (5–7). Four models were used to study the relationship between the patients' living arrangements and selected health events. No adjustments were made for Model 1. In Model 2, we adjusted for age, sex, race, and blood glucose control strategy. In Model 3, we included the parameters of Model 2 and the following conditions and diseases: smoking, previous CVD, previous heart failure, proteinuria, and depression. In Model 4, we included the parameters of Model 3 and laboratory test results for HbA1C, glomerular filtration rate (GFR), low-density lipoprotein (LDL), high-density lipoprotein (HDL), systolic blood pressure, and diastolic blood pressure, and medications including statins, aspirin, insulin, metformin, and thiazolidinedione. We did not find any evidence of violation of the proportional hazard assumption based on tests using Schoenfeld residuals. The discrimination of the models was assessed using the Harrell's C statistics. To explore the potential correlations between living arrangements and hypoglycemia risk, we compared the levels of fasting blood glucose and HbA1C between participants living alone and living with others during the follow-up period (every 4-week interval).

The propensity score matching was used to verify the association between the patients' living arrangements and selected health events in a sensitivity analysis. We used 1:1 nearest-neighbor matching without replacement and a matching tolerance (caliper) of 0.05 to match all the baseline characteristics as listed in Table 1. The propensity score was calculated using a logistic regression model, with living arrangements (dichotomized as living alone or living with others) as the dependent variable and all the potential confounders listed in Table 1 as explanatory variables. Interaction and stratified analyses by age (<60 and ≥60 years), sex, race, blood glucose control strategy (intensive or standard), smoking status, and depression were also conducted to verify the robustness of the results. We further investigated whether smoking cessation affects the association between living arrangement and our predefined outcomes. All statistical analyses were two-sided. A *P*-value of <0.05 was considered statistically significant. All analyses were performed using the STATA 15.1 (StataCorp).

**Table 1:** Baseline characteristics of included participants.

	**Live alone**		
	**Yes**	**No**	***P*-value**
***N***	**2,078**	**8,171**	
Age (years; mean ± SD)	63.71 ± 6.81	62.58 ± 6.59	<0.01
Men	976 (46.97%)	5,323 (65.15%)	<0.01
**Race**			<0.01
White	1,215 (58.47%)	5,177 (63.36%)	
Non-White	863 (41.53%)	2,994 (36.64%)	
**Glycemia control**			0.68
Intensive	1,047 (50.38%)	4,075 (49.87%)	
Standard	1,031 (49.62%)	4,096 (50.13%)	
**Smoking**			0.01
Current	323 (15.54%)	1,106 (13.54%)	
Never	888(42.73%)	3,416 (47.83%)	
Hear failure	108 (5.20%)	386 (4.72%)	0.37
CVD history	666 (32.05%)	2,942 (36.01%)	<0.01
Depression	622 (29.95%)	1,797 (22.00%)	<0.01
**Education**			0.66
Less than high school	302 (14.55%)	1,219 (14.93%)	
High-school graduate	535 (25.77%)	2,169 (26.56%)	
Some college	704 (33.91%)	2,653 (32.48%)	
College degree or higher	535 (25.77%)	2,126 (26.03%)	
Duration of diabetes	10.93 ± 7.89	10.77 ± 7.52	0.39
**(years; mean** **±SD)**			
Insurance	1,768(85.08%)	7,017(85.89%)	0.18
BMI (kg/m^2^; mean ± SD)	32.69 ± 5.59	32.11 ± 5.36	<0.01
Lipid (mg/dl; mean ± SD)			
CHOL	186.56 ± 42.50	182.47 ± 41.64	<0.01
TRIG	188.26 ± 165.30	190.61 ± 143.81	0.52
VLDL	35.95 ± 24.69	36.69 ± 24.27	0.22
LDL	106.97 ± 34.79	104.37 ± 33.67	<0.01
HDL	43.63 ± 12.80	41.42 ± 11.25	<0.01
SBP (mmHg; mean ± SD)	136.56 ± 17.66	136.31 ± 16.97	0.56
DBP (mmHg; mean ± SD)	74.83 ± 10.96	74.89 ± 10.58	0.82
HR (beats/min; mean ± SD)	73.91 ± 12.33	72.35 ± 11.58	<0.01
GFR (ml/min/1.73 m^2^)	89.41 ± 25.69	91.53 ± 27.50	<0.01
HBA1C (%; mean ± SD)	8.35 ± 0.023	8.29 ± 0.012	<0.01
**Medications**			
Aspirin	1,123 (54.49%)	4,456 (54.74%)	0.84
Statin	1,261 (61.12%)	5,238 (64.30%)	<0.01
ACEI	1,126 (54.42%)	4,442 (54.48%)	0.96
Regular insulin	233 (11.21%)	910 (11.14%)	0.92
Metformin	1,276 (61.41%)	5,277 (64.59%)	<0.01
Beta-blocker	598 (28.90%)	2,481 (30.43%)	0.18
Thiazolidinedione	392 (18.86%)	1,866 (22.84%)	<0.01

*CVD, cardiovascular disease; BMI, body mass index; CHOL, cholesterol; TRIG, triglycerides; VLDL, very low density lipoprotein; LDL, Low density lipoprotein; HDL, high density lipoprotein; SBP, systolic blood pressure; DBP, diastolic blood pressure; HR, heart rate; GFR, glomerular filtration rate; ACEI, angiotensin converting enzyme inhibitor*.

## Results

This study included 10,251 participants, 2,078 of whom lived alone; two participants were excluded because of missing information about their living status. The baseline characteristics of the participants are shown in Table 1. Compared with the participants living with others, there was a significant positive association for those living alone with the following characteristics: older age; female; non-white race; current smoker; depression; lower history of CVD; higher BMI; higher heart rate; higher levels of LDL, HDL, and HbA1C; and a lower proportion of statins, metformin, and thiazolidinedione treatment.

**Figure 1:**
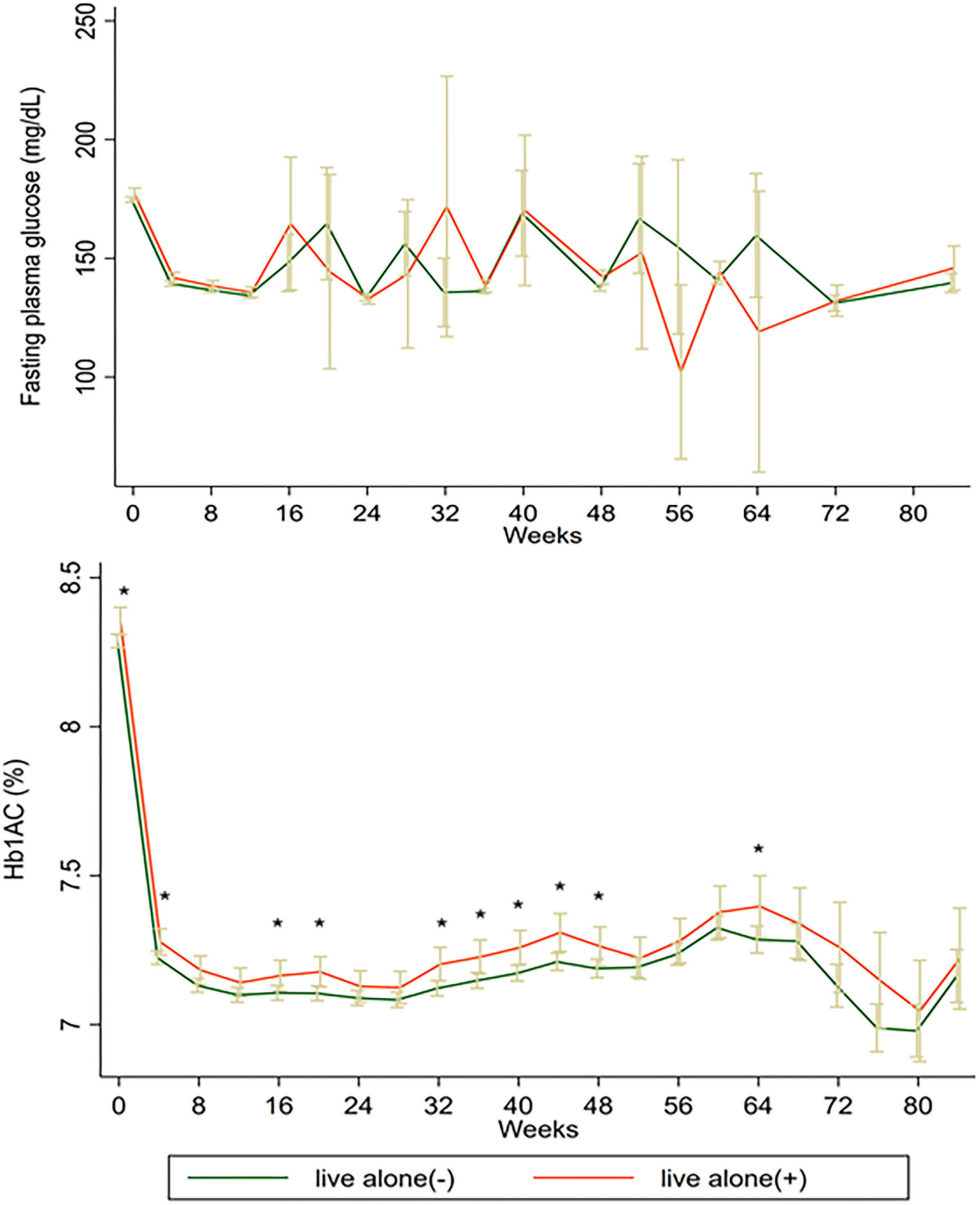
The levels of FPG and HbA1C of participants living alone and living with others. FPG, fasting plasma glucose; HbA1C, glycated hemoglobin.

The mean follow-up period was 4.91 ± 1.22 years. During follow-up, 1,046 participants developed MACEs (cardiac mortality in 331 participants, non-fatal MI in 631 participants, and non-fatal stroke in 176 participants). The rates of HMA were significantly higher in participants living alone than in those living with others (21.2 events per 1,000 person-years vs. 17.2 events per 1,000 person-years, *P* = 0.01, Table 2). The rates of HAA and MACEs did not significantly differ between participants living alone and those living with others (23.5 events per 1,000 person-years vs. 21.6 events per 1,000 person-years for MACEs; and 27.4 events per 1,000 person-years vs. 27.6 events per 1,000 person-years for HAA; Table 2). The unadjusted risk of HAA and MACEs did not significantly differ between participants living alone and those living with others (HMA, unadjusted hazard ratio (HR): 0.99, 95% CI: 0.86–1.14, *P* = 0.87; MACEs, unadjusted HR: 1.12, 95% CI: 0.96–1.30, *P* = 0.30; Table 2). Even after multivariable adjustment (Model 4), the risk of HAA, HMA, and MACEs did not differ significantly among participants living alone compared with those living with others (HAA, HR: 0.88, 95% CI: 0.75–1.04, *P* = 0.13; HMA, HR: 1.11, 95% CI: 0.92–1.34, *P* = 0.26; MACEs, HR: 0.98, 95% CI: 0.80–1.19, *P* = 0.82; Table 2). The C-index for Model 4 was 0.70 for HAA, 0.71 for HMA, and 0.67 for MACEs. As shown in Figure 1, the levels of fasting plasma glucose (FPG) did not differ during the follow-up period. However, the levels of HbA1C were higher in participants living alone than in those living with others in the middle follow-up period.

**Table 2:** Association between living alone and risk of HAA, HMA, and MACEs.

	**Live alone**		***P*-value**
**Status (*n*)**	**No (8,171)**	**Yes (2,078)**	
**MACEs**
Event rate*	21.6	23.5	
Model 1	1 (Ref)	1.12(0.96–1.30)	0.30
Model 2	1 (Ref)	1.17(1.04–1.31)	0.15
Model 3	1 (Ref)	1.02(0.87–1.21)	0.79
Model 4	1 (Ref)	0.98(0.80–1.19)	0.82
**HAA**
Event rate*	27.6	27.4	
Model 1	1 (Ref)	0.99(0.86–1.14)	0.87
Model 2	1 (Ref)	0.90(0.78–1.03)	0.13
Model 3	1 (Ref)	0.88(0.75–1.02)	0.09
Model 4	1 (Ref)	0.88(0.75–1.04)	0.13
**HMA**
Event rate*	17.2	21.2	
Model 1	1 (Ref)	1.23(1.05–1.44)	0.01
Model 2	1 (Ref)	1.10(0.94–1.32)	0.13
Model 3	1 (Ref)	1.10(0.91–1.30	0.33
Model 4	1 (Ref)	1.11(0.92–1.34)	0.26

* *per 1,000 person-years*.

*Model 1: unadjusted; Model 2: adjusted for age, sex, blood glucose control strategy, race; Model 3 adjusted for age, sex, blood glucose control strategy, race, education, insurance, smoking, previous cardiovascular disease, previous heart failure, proteinuria, depression; model 4: model 3 in addition to HbA1C, glomerular filtration rate, LDL, SBP, DBP, BMI, statin, aspirin, insulin, metformin, thiazolidinedione. Ref: reference*.

*HAA, hypoglycemia requiring any assistance; HMA, hypoglycemia requiring medical assistance*.

Participants living alone showed a similar risk of cardiac death, non-fatal MI, and non-fatal stroke as those living with others (cardiac death, HR: 1.13, 95% CI: 0.74–1.76, *P* = 0.56, Model 4; non-fatal MI, HR: 0.92, 95% CI: 0.73–1.17, *P* = 0.49, Model 4; non-fatal stroke, HR: 0.92, 95% CI: 0.57–1.46, *P* = 0.71, Model 4, Table 3).

**Table 3:** Association between living alone and individual components of MACEs.

	**Live alone**		***P*-value**
**Status(n)**	**No (8,171)**	**Yes (2,078)**	
**Cardiac death**
Event rate^*^	6.4	7.5	
Model 1	1 (Ref)	1.16(0.90-1.50)	0.26
Model 2	1 (Ref)	1.19(0.91-1.54)	0.21
Model 3	1 (Ref)	1.16(0.88-1.54)	0.30
Model 4	1 (Ref)	1.13(0.74-1.76)	0.56
**Non-fatal MI**
Event rate^*^	13.2	13.4	
Model 1	1 (Ref)	1.02(0.84-1.24)	0.85
Model 2	1 (Ref)	1.03(0.88-1.30)	0.52
Model 3	1 (Ref)	0.96(0.77-1.20)	0.72
Model 4	1 (Ref)	0.92(0.73-1.17)	0.49
**Non-fatal stroke**
Event rate^*^	3.4	4.1	
Model 1	1 (Ref)	1.17(0.82-1.67)	0.39
Model 2	1 (Ref)	1.18(0.82-1.68)	0.37
Model 3	1 (Ref)	0.96(0.67-1.33)	0.86
Model 4	1 (Ref)	0.92(0.57-1.46)	0.71

* *per 1,000 person-years*.

*Model 1: unadjusted; Model 2: adjusted for age, sex, blood glucose control strategy, race; Model 3 adjusted for age, sex, blood glucose control strategy, race, education, insurance, smoking, previous cardiovascular disease, previous heart failure, proteinuria, depression; model 4: model 3 in addition to HbA1C, glomerular filtration rate, LDL, SBP, DBP, BMI, statin, aspirin, insulin, metformin, thiazolidinedione. Ref: reference*.

*HbA1C, Glycosylated hemoglobin; GFR, glomerular filtration rate; LDL, Low density lipoprotein; HDL, high density lipoprotein; SBP, systolic blood pressure; DBP, diastolic blood pressure*.

**Figure 2:**
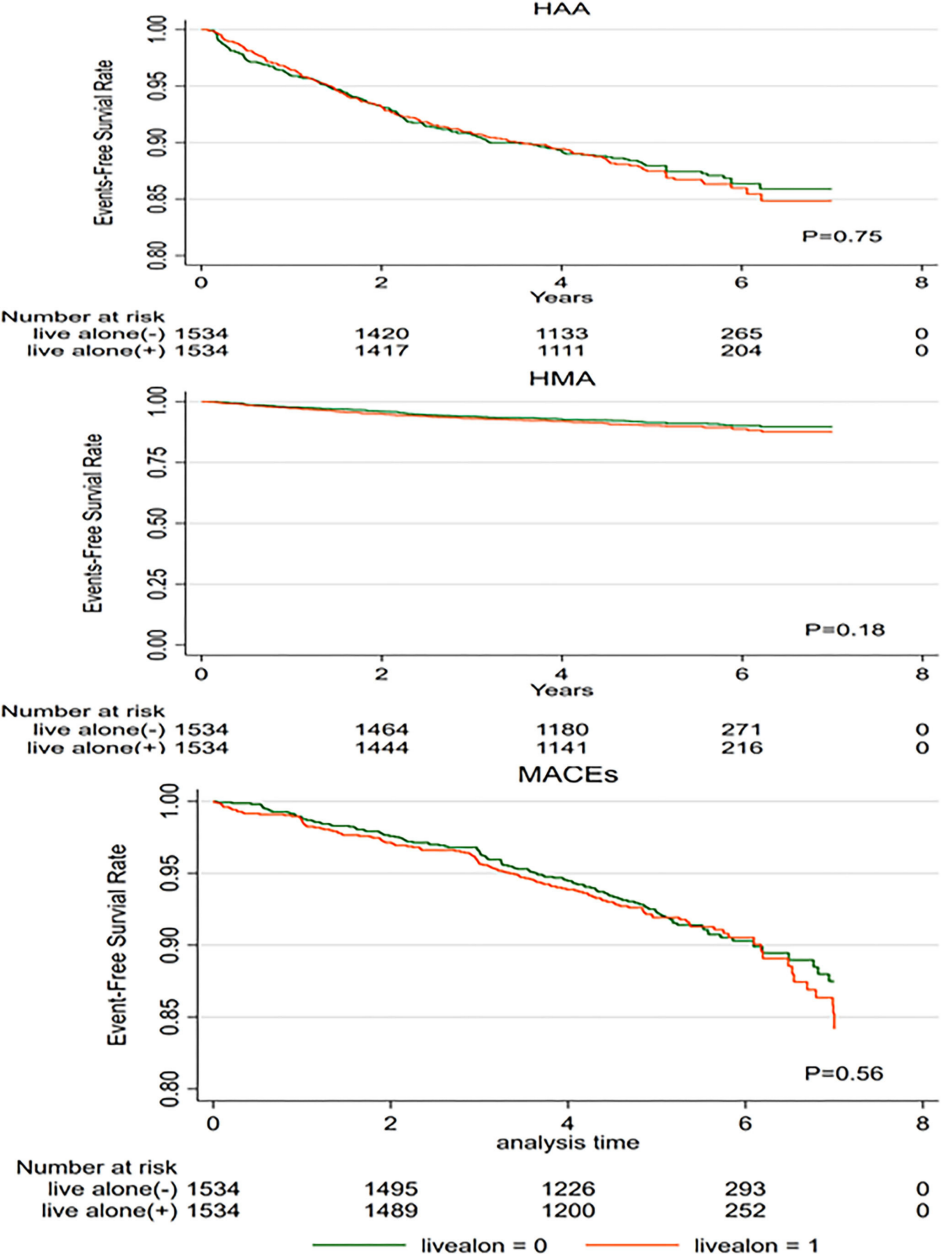
Kaplan–Meier survival curves for the selected health events in propensity score-matched patients with type 2 diabetes. MACEs, major adverse cardiovascular events; HAA, hypoglycemia requiring any assistance; HMA, hypoglycemia requiring medical assistance.

**Figure 3:**
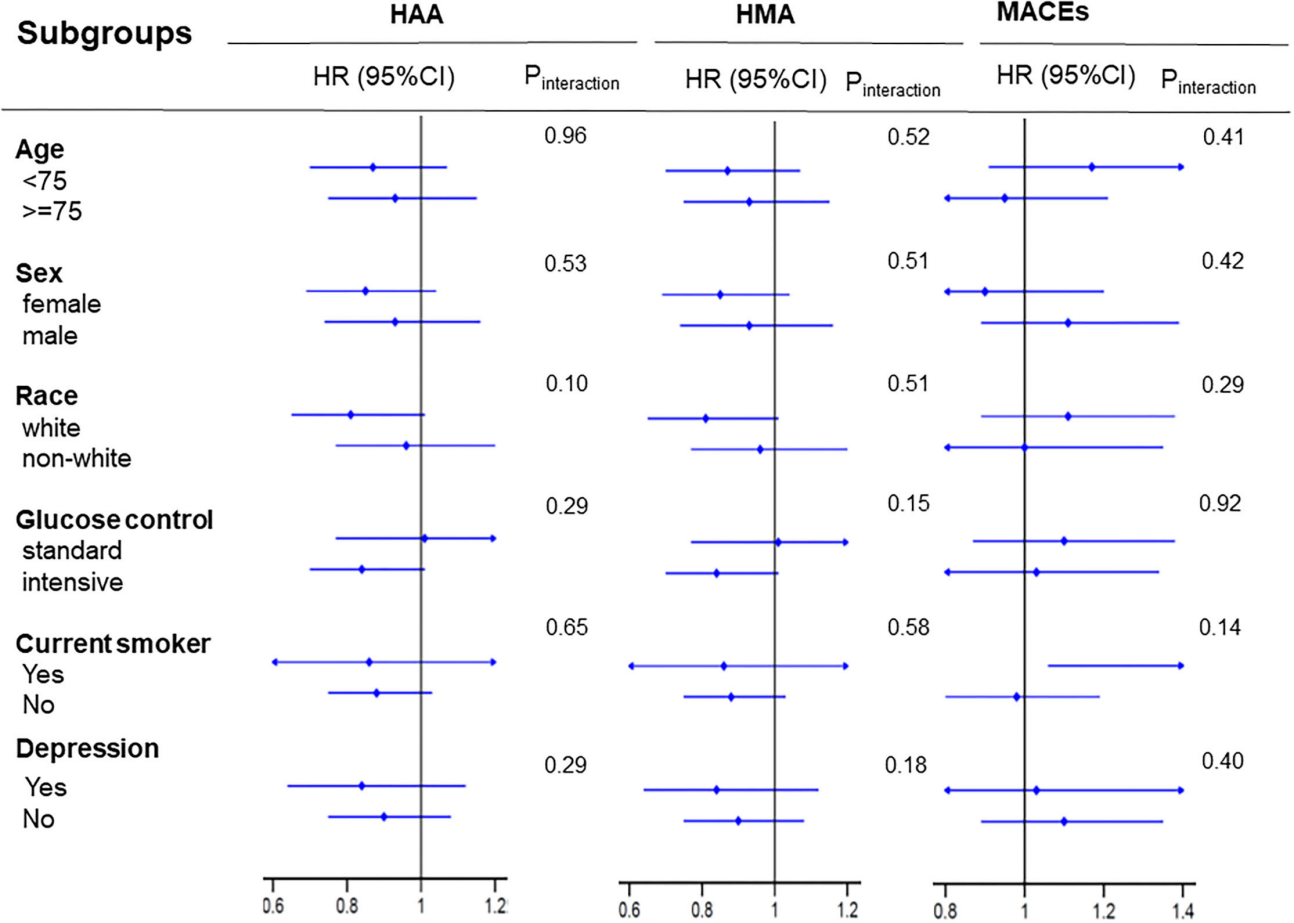
Subgroup analysis for the selected health events. Each stratification was adjusted for all factors in Model 4, except for the stratification factor itself. MACEs, major adverse cardiovascular events; HAA, hypoglycemia requiring any assistance; HMA, hypoglycemia requiring medical assistance.

We performed additional sensitivity analyses to verify the association between living alone and the selected health events in patients with T2DM using propensity score matching. Baseline characteristics between the participants living alone and those living with others did not differ significantly (data did not show); this was same for the HAA risk, HMA risk, and risk of MACEs (Figure 2). We also performed subgroup and interactive analyses to assess the robustness of the association between living alone and the selected health events. Figure 3 shows the association between living alone and the selected health events in the different subgroups. Although we did not find a significant interactive effect of living alone and smoking status on MACEs, a higher risk of MACEs was observed in current smokers living alone than in those living with others (HR: 1.6, 95% CI: 1.06–2.42, *P* < 0.01). When compared to the never smokers, participants living alone who had quit smoking did not have higher risk of MACE than those living with others.

## Discussion

In this *post-hoc* analysis of the ACCORD study (9), we found that living alone did not increase the risk of HAA, HMA, or MACEs, including cardiac death, non-fatal MI, and non-fatal stroke, among patients with T2DM.

Living alone has become an important welfare issue for older people in rapidly aging societies. Although previous studies have found that living alone is associated with an increased risk of CVD, little is known about the effect of living alone in older type 2 diabetes patients on clinical outcomes, especially hypoglycemia. Intensified glucose control may lead to a higher incidence of hypoglycemia and higher MACEs (11). Although the levels of FPG did not differ between participants living alone and those living with others, participants living alone had higher levels of HbA1C. A less aggressive glucose control strategy may be one of the reasons that patients with T2DM living alone did not have a higher risk of hypoglycemia or MACEs. Previous studies have found that patients living alone may have more difficulties in social support and experience more negative emotions, such as depression and loneliness (12). However, older persons living alone might be considered more likely to have a good physical condition, particularly in terms of independence in activities of daily living, which is a powerful indicator of a lower incidence of hypoglycemia and CVD (12, 13). These two factors might have a directionally opposite impact on the association between living alone and the incidence of hypoglycemia and CVD. The baseline characteristics of the included participants, covering different extents of the above two factors, may explain the unexpected results. Some studies have found that older people living alone were more likely to have a good functional status, which decreased the need for social support (14–16). All included participants were recruited from the United States and Canada; these countries have a better public health insurance system and higher income than developing counties. This may also explain the association of living alone with higher MACEs and hypoglycemia. Furthermore, like in a previous study (17), we found that a larger proportion of patients living alone among the elderly was female, unlike the sex distribution among younger patients living alone. Women were more likely to have good functional status and were less likely to develop adverse events than men (18).

Smokers living alone were more likely to develop MACEs because smokers living alone smoked more cigarettes than those living with others (19). Complex factors associated with living alone may explain why recent studies have reported discordant results regarding the outcomes of patients who live alone. Therefore, we suggest that more attention should be paid to cardiovascular risk factors and administration of hypoglycemic drugs rather than on the living arrangement itself.

Our findings have several potential clinical implications. Patients living alone had higher Hb1AC levels than those living with others. Therefore, clinicians should consider an effective blood glucose control regardless of their living arrangement through education and network-based guidance. Patients living alone tend to smoke more and thus have a higher risk of MACEs. Social support and education are needed to help them quit smoking. This study had some limitations. First, we did not analyze any data related to changes in the living arrangements during follow-up, and we cannot comment on whether the risk of CVD and hypoglycemia is dynamic over time. Second, the follow-up period was short (4.91 ± 1.22 years), which may have affected our results. Third, we omitted socioeconomic data such as employment status, income, diet, and physical activity. This socioeconomic information may have affected our results. Finally, all included patients were from the United States and Canada, and the findings of this study may not be applicable to other populations.

## Conclusion

In patients with T2DM, living alone did not increase the risk of cardiovascular events (cardiac death, non-fatal MI, or non-fatal stroke) and hypoglycemia. Patients living alone had higher Hb1AC levels than those living with others. Clinicians should consider an effective blood glucose control regardless of their living arrangement.

## Data Availability Statement

The original contributions presented in the study are included in the article/supplementary materials, further inquiries can be directed to the corresponding author/s.

## Ethics Statement

The studies involving human participants were reviewed and approved by NIH. The patients/participants provided their written informed consent to participate in this study.

## Author Contributions

ZZ and ZP drafted the manuscript. ZX is the guarantor of this work, had full access to all the data in the study, takes responsibility for the integrity of the data, the accuracy of the data analysis, designed the study, and provided methodological expertise. All authors have read, provided critical feedback on, and approved the final manuscript.

## Funding

This work was supported in part by National Science Foundation of China project 82000298 and Natural Science Foundation of Hunan Province 2021JJ40883 to ZX.

## Conflict of Interest

The authors declare that the research was conducted in the absence of any commercial or financial relationships that could be construed as a potential conflict of interest.

## Publisher's Note

All claims expressed in this article are solely those of the authors and do not necessarily represent those of their affiliated organizations, or those of the publisher, the editors and the reviewers. Any product that may be evaluated in this article, or claim that may be made by its manufacturer, is not guaranteed or endorsed by the publisher.
